# Tomato yellow leaf curl virus: No evidence for replication in the insect vector *Bemisia tabaci*

**DOI:** 10.1038/srep30942

**Published:** 2016-08-01

**Authors:** Sonia Sánchez-Campos, Edgar A. Rodríguez-Negrete, Lucía Cruzado, Ana Grande-Pérez, Eduardo R. Bejarano, Jesús Navas-Castillo, Enrique Moriones

**Affiliations:** 1 Instituto de Hortofruticultura Subtropical y Mediterránea “La Mayora” (IHSM-UMA-CSIC), Universidad de Málaga - Consejo Superior de Investigaciones Científicas, Estación Experimental “La Mayora”, 29750 Algarrobo-Costa, Málaga, Spain; 2 Instituto de Hortofruticultura Subtropical y Mediterránea “La Mayora” (IHSM-UMA-CSIC), Universidad de Málaga - Consejo Superior de Investigaciones Científicas, Área de Genética, Campus de Teatinos, 29071 Málaga, Spain

## Abstract

Begomovirus ssDNA plant virus (family *Geminiviridae*) replication within the *Bemisia tabaci* vector is controversial. Transovarial transmission, alteration to whitefly biology, or detection of viral transcripts in the vector are proposed as indirect evidence of replication of tomato yellow leaf curl virus (TYLCV). Recently, contrasting direct evidence has been reported regarding the capacity of TYLCV to replicate within individuals of *B. tabaci* based on quantitave PCR approaches. Time-course experiments to quantify complementary and virion sense viral nucleic acid accumulation within *B. tabaci* using a recently implemented two step qPCR procedure revealed that viral DNA quantities did not increase for time points up to 96 hours after acquisition of the virus. Our findings do not support a recent report claiming TYLCV replication in individuals of *B. tabaci*.

Plant viruses are economically important pathogens and most rely on insect vectors for their transmission from plant-to-plant, which is an essential step of their life cycle. Among them, begomoviruses (genus *Begomovirus*, family *Geminiviridae*) is an emergent group of single-stranded circular DNA viruses that comprise some of the most important plant viruses that cause serious harm to food and fiber crops in the world^1^.

Understanding the transmission processes might help to design more effective control strategies of plant viruses through interference with vector transmission; basic information on the virus-vector interaction process, however, is scarce for many plant viruses^2^. Numerous insect vectored plant viruses are transmitted in a non-persistent or semipersistent non-circulative manner with the virus maintained only temporarily in the mouthparts or foregut of the vector without circulating through its body; however, a significant number is transmitted in a persistent circulative manner in which the viruses cross the gut epithelium and circulate through the body of the insect vector to ultimately colonize the salivary glands before being transmitted to plants during feeding^3^. In the latter case, some viruses can replicate within the vector and are called propagative for which transmission can be maintained even during the entire lifespan of the insect vector with dramatic consequences for the epidemiology and control of the virus^4^.

Begomoviruses are transmitted in a circulative manner by their whitefly (Hemiptera: Aleyrodidae) insect vector *Bemisia tabaci*^2^,^5^. These viruses consist of circular single-stranded DNA (ssDNA) packaged into twin-shaped (geminate) icosahedral particles^6^. After begomovirus transmission to the plant, the viral ssDNA is released from the virus particle and converted to the double-stranded DNA (dsDNA) replicative form. Then, through rolling circle replication (RCR), complementary-sense (CS) and virion-sense (VS) DNA strands are produced with the help of the plant enzyme machinery and the virus replication-associated protein (Rep)^7^,^8^. Therefore, presence of CS DNA can be used to support the occurrence of virus replication. Finally, VS ssDNA circles are encapsidated into new viral particles that can be acquired and transmitted to healthy plants by individuals of the *B. tabaci* vector.

For decades, begomoviruses and other members of the *Geminiviridae* were thought to be acquired and circulated in their insect vectors without replicating, yet can be transmitted for essentially the lifetime of the vector. Only very recently with the advent of highly refined quantitative PCR methods it has come into question whether replication of begomoviruses may also occur in the *B. tabaci*,vector. Currently, controversy exists about the possible ability of these viruses to replicate in their insect vector. Indirect evidence has been provided in this sense by several reports suggesting replication of the monopartite begomovirus tomato yellow leaf curl virus (TYLCV). Thus, following acquisition, TYLCV is retained by the whitefly vector for its entire lifespan. Transovarial transmission and alteration of the whitefly biology have been described, and a transient increase in TYLCV DNA was detected for the Middle East-Asia Minor 1 (MEAM1) species (formerly B biotype) of the *B. tabaci* cryptic complex^9^,^10^,^11^,^12^,^13^. Moreover, the accumulation of viral transcripts of genes located in either TYLCV VS or CS DNA strands was detected in *B. tabaci* using standard or real time RT-PCR or RNA probes suggestive of the presence in the insect of the dsDNA replicative forms^10^,^14^,^15^. However, recently, contrasting direct evidence has been reported regarding the capacity of TYLCV to replicate within individuals of the MEAM1 species based on quantitave PCR approaches. Thus, results obtained by Pakkianathan *et al.*^16^ seem to support replication of an Israeli isolate of TYLCV (a strain not specified but presumably belonging to the strain Israel, IL) within the insect vector. In contrast, no such replication was observed by Becker *et al.*^17^ for isolates of the Mild (TYLC-Mld) and IL (TYLCV-IL) strains of TYLCV.

In the present study we contribute to the debate on whether TYLCV can replicate in *B. tabaci* by providing data from direct quantitative analysis of viral CS and VS DNA strands accumulation within *B. tabaci* individuals using a recently developed sensitive and strand-specific amplification procedure^18^. Analyses conducted by Pakkianathan *et al.*^16^ and Becker *et al.*^17^ were performed on *B. tabaci* individuals maintained on TYLCV non-host plants until they were analyzed after the virus acquisition access period (AAP). Here, we assess the ability of TYLCV to replicate in the insect in the absence of plant factors that could complement virus replication by feeding the whiteflies on preparations of purified virions during the virus AAP and maintaining them on an artificial diet after the AAP. As transmission of TYLCV might be affected by the *B. tabaci* genotype^19^,^20^, the kinetics of CS and VS DNA strands accumulation after the AAP was monitored for the two major invasive species of the cryptic *B. tabaci* complex, MEAM1 and Mediterranean (MED, formerly the Q biotype). Also, representatives of two emerging TYLCV strains, TYLC-Mld and TYLCV-IL, were included in the study. Our results showed that CS and VS DNA loads in whiteflies remained stable or slightly decreased post-AAP, consistent with the absence of TYLCV replication in *B. tabaci*. Since TYLCV ranks among the top ten most devastating plant viruses^1^,^21^, precise knowledge about virus-insect interaction, especially replication in the insect, might help to improve its control.

## Results and Discussion

Replication of isolates of the Israel and Mild strains of TYLCV (TYLCV-IL and TYLCV-Mld, respectively) was analyzed in individuals of MEAM1 and MED species of the *B. tabaci* cryptic complex^11^. These two TYLCVs and the two whitefly species that differ genetically and biologically^5^,^13^,^22^ were used to explore different biological combinations. TYLCV purified virions were acquired by whiteflies on an artificial sucrose diet in an attempt to provide them only encapsidated virus VS forms. Thus, acquisition from plants as done in previous studies^10^,^15^,^17^ was not performed to diminish or prevent the acquisition of CS forms and/or viral transcription and/or replication complexes that could be present in infected plants. Also, previous to virus acquisition, purified virions were treated with DNase I to eliminate or reduce the presence of non-encapsidated viral nucleic acids. Moreover, after AAP and until analysis, whiteflies were maintained on an artificial diet and not on a virus non-host plants as done in previous studies^16^,^17^ to eliminate plant factors that could help to complete the virus life cycle within the whiteflies. The infectivity of purified-DNase I-treated virions was confirmed through *B. tabaci* transmission to tomato plants. Analysis of test plants for TYLCV infection at 21 days post *B. tabaci*-inoculation indicated that transmission from purified and DNase I-treated virion preparations was achieved by both *B. tabaci* MEAM1 (9 and 5 plants infected out of 15 inoculated for TYLCV-IL and TYLCV-Mld, respectively) or MED (2 out of 15 both for TYLCV-IL and TYLCV-Mld), resulting in symptomatic infections. Therefore, biologically active virion preparations were provided to whiteflies.

**Figure 1:**
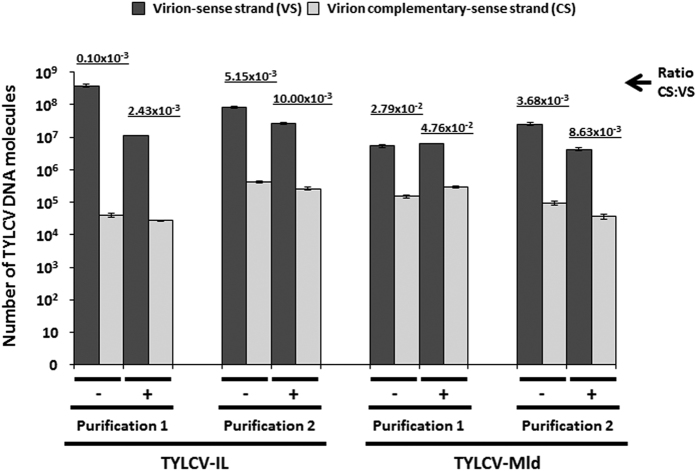
Quantification of tomato yellow leaf curl virus sense and complementary sense strand DNA molecules in virus purifications. Number of virus sense (VS, dark grey bars) and complementary-sense (CS, light grey bars) strand DNA molecules of tomato yellow leaf curl virus (TYLCV) quantified in 10 μl of TYLCV virion purifications of the Israel (TYLCV-IL) and Mild (TYLCV-Mld) strains before (−) and after (+) DNase I treatment. These virus purifications were used for *B. tabaci* feeding in this work to determine if virus replication occurs within them. As the presence of CS strands is indicative of virus replication, highlighting their occurrence in the virus diet provided to whiteflies is important. Two independent virus purifications per virus strain were obtained and analyzed. Quantification of VS and CS DNA molecules was performed according to Rodríguez-Negrete *et al.*^18^. Data represent the mean and standard error of three independent qPCR technical replicates. The CS:VS ratio for each virus purification is indicated. PCR efficiency values measured across a dynamic range of 10^3^ to 10^9^ template molecules were 95.73% for TYLCV-Mld and 95.06% for TYLCV-IL in the case of VS detection. Efficiency values in CS quantification were 95.44% for TYLCV-Mld and 95.17% for TYLCV-IL.

Quantification of the number of VS and CS TYLCV DNA molecules present in purified TYLCV virion preparations before and after DNase I treatment (Fig. 1) showed that i) both VS and CS viral DNAs could be detected in those preparations and ii) DNase I treatment helped to reduce but did not completely eliminate non-encapsidated DNA within the preparations, with a decreased amount of CS molecules detected in three out of four treated preparations. A decrease in the number of VS DNA molecules was also observed in the same treated preparation, probably due to degradation of co-purified but non-encapsidated VS DNA molecules.

**Figure 2:**
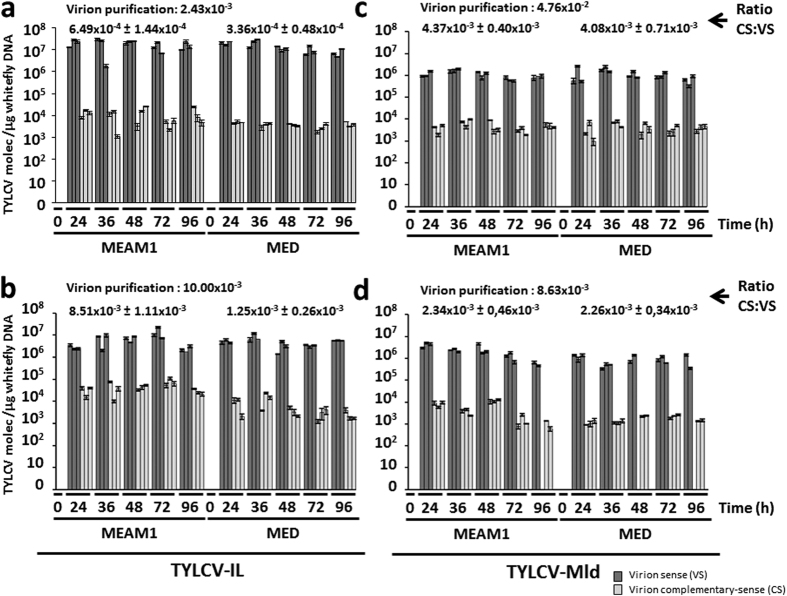
Dynamics of tomato yellow leaf curl virus sense and complementary sense strand DNA molecules accumulation in *Bemisia tabaci* individuals. Time-course analysis of the amount of tomato yellow leaf curl virus (TYLCV) virus sense (VS, dark grey bars) and complementary-sense (CS, light grey bars) strand DNA molecules present in individuals of the Middle East-Asia Minor 1 (MEAM1) and Mediterranean (MED) species of *Bemisia tabaci* that were given a 24 h acquisition access period (AAP) of TYLCV Israel (TYLCV-IL) (two replicated experiments, panels a,b) or Mild (TYLCV-Mld) (two replicated experiments panels c,d) strains virion purifications. Acquisition was done in an *in vitro* feeding (artificial diet) experiment and whiteflies were maintained on an artificial diet after virus acquisition until analysis. The amounts of CS and VS were quantified at 0, 24, 36, 48, 72, and 96 hours after the beginning of the AAP from DNA extracted from 50 whitefly individuals (values for three biological replicates per time point, for each *B. tabaci* genotype and virus combination are shown). The mean CS:VS ratio (±standard error) for each virus and whitefly genotype combination through each time-course study is indicated; CS:VS ratio in virion purifications used for virus acquisition is also indicated. Quantification of VS and CS molecules was performed on DNA extracts according to Rodríguez-Negrete *et al.*^18^. Data from insect extracts were normalized to the 18S ribosomal RNA gene. The mean and standard error of three independent qPCR technical replicates are shown. PCR efficiency values along a dynamic range of 10^3^ to 10^9^ molecules were 94.13% for TYLCV-Mld and 94.6% for TYLCV-IL for VS detection. Efficiency values for CS measurements were 96.48% for TYLCV-Mld and 95.15% for TYLCV-IL.

The kinetics (0, 24, 36, 48, 72, and 96 hours) of viral VS and CS DNA molecules accumulation within *B. tabaci* whiteflies after the AAP on virion preparations are summarized in Fig. 2. Both VS and CS DNA molecules were detected in whiteflies as expected for acquisition from sources in which both types of molecules were present. Therefore, in addition to virions, whiteflies could acquire non-encapsidated DNA forms including CS DNA which is not present in viral particles. Similar uptake of CS DNA with the phloem sap ingested by *B. tabaci* individuals fed on TYLCV infected plants following transfer to non-host plant has been shown either by primer extension followed by Southern blotting^10^ or by real-time PCR^17^. However, a comparison of CS:VS ratios from whiteflies or virion purifications used as the virus source (Fig. 2), showed that VS DNA molecules concentrate in whiteflies during the acquisition process, which suggests preferential virion acquisition (that only contain VS DNA molecules) by whiteflies. Constant CS:VS ratios were detected in whiteflies throughout the time-course with no significant differences detected (one-way ANOVA and Tukey-b test, P = 0.01) except for two specific cases (MED with TYLCV-IL at 96 h and MEAM1 with TYLCV-Mld at 48 h in replication 1 and 2, respectively (Supplementary Table S1). Whether a tendency existed over time for the accumulation of VS and/or CS molecules in whiteflies was tested by linear contrasts within ANOVA (see Methods). No significant linear tendency (P = 0.01) was observed in most combinations of the *B. tabaci* genotype vs. TYLCV strain (Supplementary Table S2), with other level contrasts (quadratic, cubic, etc.) also indicating no significant trend (data not shown). A significant linear tendency was detected only in four cases: three for VS (MED with TYLCV-IL, assay in panel a, and MEAM1 with TYLCV-Mld, assays in panels c and d, Fig. 2), and one for CS (MEAM1 with TYLCV-Mld, assay in panel d, Fig. 2). Nevertheless, even in those cases, the slopes of the regression lines were negative and close to zero (Supplementary Table S2), suggesting slight degradation rather than accumulation. It is worth mentioning that a similar progressive decrease in the quantity of CS DNA in MEAM1 *B. tabaci* individuals was detected by Becker *et al.*^17^ after AAP for the Mld and IL strains of TYLCV, also supporting degradation. In this sense, the decrease in TYLCV DNA amounts in *B. tabaci* MEAM1 whiteflies after AAP detected by Pakkianathan *et al.*^16^ in comparative time lapses while maintained on a non-host plant was attributed to destruction by the innate whitefly immune system activated against the virus. Although Pakkianathan *et al.*^16^ suggested virus DNA replication in the insect vector because of increased amounts of viral RNA transcripts (including transcripts from CS DNA strands) detected during the first 1 to 5 days post-AAP when fed on a non-host plant, no such a replication could be supported either by our results or by those of Becker *et al.*^17^. It should be stressed that it cannot be discarded that viral DNA transcription from CS DNA strands detected by Pakkianathan *et al.*^16^ could derive from TYLCV CS DNA acquired by whiteflies from virus source plants during the AAP and not as a result of virus replication. Acquisition of increased amounts of CS DNA during AAP and persistence in *B. tabaci* individuals until 20 days post-AAP was demonstrated by Becker *et al.*^17^ for the IL and the Mld strains of TYLCV. In this sense, the persistence of nucleic acids in *B. tabaci* for several days after acquisition from a feeding source has been previously demonstrated^23^.

Therefore, under the experimental conditions used here (virus acquisition on virion preparations and maintenance of whiteflies post-AAP on an artificial diet until VS and CS strands quantification), constant CS:VS ratios and no significant increase trend in CS or VS TYLCV DNA strands accumulation shown here suggests the persistence of acquired VS or CS molecules, with no evidence for TYLCV replication in *B. tabaci* individuals. Similar results were obtained for TYLCV-Mld or TYLCV-IL with either the MEAM1 or the MED species of *B. tabaci*. In contrast, increased virus accumulation in the insect vector in time-frames similar to those used here was shown for propagative plant RNA viruses of the genus *Tospovirus* (family *Bunyaviridae*) in thrips^24^,^25^,^26^ or *Tenuivirus* in planthoppers^27^. More than a ten-fold titer increase was observed for the plant RNA and aphid-borne virus raspberry latent virus (family *Reoviridae*) in its insect vector at five days post-AAP^28^. Similarly, for circulative propagative transmission frequent among arthropod-borne viruses transmitted to vertebrates, even 100-fold increase in viral load has been observed using quantitative methods during the first days post-AAP, such as for the dengue virus (family *Flaviviridae*) transmitted by the mosquito *Aedes albopictus*^29^. A slight increase in TYLCV load in *B. tabaci* individuals during the first 1 to 5 days post-AAP was described by Pakkianathan *et al.*^16^, suggesting replication. However, no such increase was detected by Becker *et al.*^17^ using a similar experimental procedure, nor in the present study when feeding whiteflies an artificial diet to eliminate plant factor interference. Therefore, if TYLCV replicates in the whitefly vectors, the rate is not very high and is far lower than that of the other propagative viruses mentioned here.

Thus, using a different experimental approach, no evidence was obtained for substantial replication of TYLCV-Mld or TYLCV-IL in individuals of either the MEAM1 or MED species of *B. tabaci*, in agreement with the results obtained by Becker *et al.*^17^. We cannot discard the existence of an equilibrium between replication and degradation or the prevention of virus accumulation by insect innate immune responses, as suggested for *B. tabaci*^16^ and for vectors of vertebrate viruses^30^,^31^. The requirement of some plant factor(/s) for TYCLV replication in *B. tabaci* that could be acquired with the phloem sap ingested during whitefly feeding on plants was not supported by the results of Becker *et al.*^17^. The effects of TYLCV on *B. tabaci* biology (survival, fertility and behavior)^10^,^13^,^32^,^33^,^34^,^35^ were claimed to be indirect evidence of TYLCV replication in the insect^9^,^10^. However, in those experiments, the virus was acquired by whiteflies from TYLCV-infected plants, and we now know that virus infection can lead to changes in plant status (e.g. Maluta *et al.*^36^) that could have indirect effects on *B. tabaci* biology. Therefore, additional experimental evidence is needed to definitively affirm or discard the replication of TYLCV in *B. tabaci*.

## Methods

### Virus isolates and virion purification

Infectious clones of isolates [ES:Alm:Pep:99] of TYLCV-IL (GenBank accession number AJ489258) and [ES:72:97] of TYLCV-Mld (GenBank AF071228) have been described previously by Morilla *et al.*^37^ and by Navas-Castillo *et al.*^38^, respectively. Virus purification was conducted from systemically infected *N. benthamiana* plants inoculated with TYLCV-IL or TYLCV-Mld infectious clones via *Agrobacterium tumefaciens*-mediated inoculation in the axillary bud of the fourth/fifth leaf of 3 to 4 week-old plants^39^. Three weeks after inoculation, virions were purified from young infected leaves of *N. benthamiana* plants following method II described by Czosnek *et al.*^40^, omitting the sucrose gradient step. Pellets containing virions were resuspended in 0.1M phosphate buffer, pH 7.0, 0.2 mM EDTA (5 μl per leaf gram extracted) and stored at −70 °C in 50% glycerol until used. Before use, preparations were treated with 1 U/μl DNase I (Roche Diagnostics, Mannheim, Germany) (28 °C, 3 h) to eliminate non-encapsidated DNA. DNAse I-treated and untreated preparations were kept at equal volumes previous to strand specific viral DNA quantification (see below).

### Whiteflies, virus acquisition and transmission

*B. tabaci* adult individuals of the MEAM1 and MED species of the *B. tabaci* cryptic complex^11^ were used for virus acquisition and transmission. For purified TYLCV acquisition, virion preparations were provided to whiteflies (24 h AAP) on an artificial diet (25% sucrose supplemented with tartrazine yellow food dye)^41^. Groups of 50 whiteflies were used to acquire TYLCV from 200 μl of virion preparations, as previously described^23^. After the AAP, whiteflies were maintained on a virion-free artificial diet until analysis or use in transmission assays. Three biological replicates consisting of 50 whiteflies each were collected per time point and whitefly-virus combination, and used for the quantification of viral VS and CS DNA forms. Each whitefly-virus combination experiment was repeated twice.

For virus transmission assays, groups of 15 whiteflies collected after the AAP were provided a 72 h inoculation access period (IAP) on tomato cv. Moneymaker (La Mayora-CSIC seed bank) plants at the three-leaf growth stage. Young non-inoculated leaves of test plants were then analyzed at 21 days post inoculation for TYLCV infection by tissue blot hybridization, following Navas-Castillo *et al.*^38^.

### Strand-specific viral DNA quantification

Viral VS and CS DNA molecules accumulation within whiteflies was monitored at 0, 24, 36, 48, 72, and 96 hours after the beginning of the AAP. We used a recently described^18^ strand-specific two-step qPCR quantification procedure to analyze VS and CS strand accumulation on virion purifications or on DNA extracts from whiteflies (50 individuals per sample) obtained using the JETFLEX Genomic DNA Purification Kit (GenoMed, Inc., Leesburg, FL). To normalize qPCR data from insects, the 18S ribosomal RNA gene of *B. tabaci* was amplified from each sample^42^.

### Statistical analyses

Mean CS:VS ratios were compared using one-way ANOVA and the Tukey-b comparison of means test (P = 0.01). Whether a tendency existed over time for the accumulation of VS and CS molecules in whiteflies was tested statistically by subjecting the log transformed data of CS and VS amounts to one-way ANOVAs by time and testing (P = 0.01) linear contrasts. Analyses were performed with the SPSS statistical package v.22.

## Additional Information

**How to cite this article**: Sánchez-Campos, S. *et al.* Tomato yellow leaf curl virus: No evidence for replication in the insect vector *Bemisia tabaci. Sci. Rep.* **6**, 30942; doi: 10.1038/srep30942 (2016).

## Supplementary Material

Supplementary Information
