# Supplementary material for: Tomato yellow leaf curl virus: No evidence for replication in the insect vector Bemisia tabaci
Source: Sci Rep. 2016 Aug 1;6:30942. doi: 10.1038/srep30942 (PMC4967916; doi:10.1038/srep30942)
Supplement: Supplementary Information [file srep30942-s1.pdf]

Tomato yellow leaf curl virus: No evidence for replication in the insect vector *Bemisia tabaci*

Sonia Sánchez-Campos, Edgar A. Rodríguez-Negrete, Lucía Cruzado, Ana Grande-Pérez, Eduardo R. Bejarano, Jesús Navas-Castillo & Enrique Moriones

Supplementary Table S1. Ratios for the amount of tomato yellow leaf curl virus (TYLCV) complementary-sense (CS) versus virus sense (VS) strand DNA molecules (CS:VS) present in individuals of the Middle East-Asia Minor 1 (MEAM1) and Mediterranean (MED) species of *Bemisia tabaci* that were given a 24 h acquisition access period (AAP) on TYLCV strain Israel (TYLCV-IL) or Mild (TYLCV-Mld) (two replicated experiments each) virion preparations and then maintained on virus-free artificial sucrose diet. CS and VS amounts were calculated from DNA extracted from 50 whitefly individuals (three biological replicates per time point for each *B. tabaci* genotype and virus combination) at 0, 24, 36, 48, 72, and 96 hours after the beginning of the AAP. Quantitative analysis of VS and CS molecules was performed on DNA extracts according to Rodríguez-Negrete et al. (1). Data from insect extracts were normalized to the 18S ribosomal RNA gene. The mean CS:VS ratio (± standard error) for each virus/whitefly genotype combination at each time-point is shown. Values significantly different (one-way ANOVA and Tukey-b test, P=0.01) in each time course are highlighted with an asterisk.

Virus isolate	*Bemisia tabaci* species	Time	Replication 1	Replication 2
			Mean CS:VS ratio ± standard error	Mean CS:VS ratio ± standard error
TYLCV-IL	MEAM1	0	-	-
		24	$5.93 \times 10^{-4} \pm 1.37 \times 10^{-5}$	$1.13 \times 10^{-2} \pm 2.88 \times 10^{-3}$
		36	$4.90 \times 10^{-4} \pm 6.51 \times 10^{-5}$	$6.05 \times 10^{-3} \pm 1.50 \times 10^{-3}$
		48	$6.68 \times 10^{-4} \pm 2.63 \times 10^{-4}$	$6.31 \times 10^{-3} \pm 1.37 \times 10^{-3}$
		72	$3.25 \times 10^{-4} \pm 2.18 \times 10^{-4}$	$5.62 \times 10^{-3} \pm 1.27 \times 10^{-3}$
		96	$7.64 \times 10^{-4} \pm 7.10 \times 10^{-4}$	$1.16 \times 10^{-2} \pm 3.08 \times 10^{-3}$
	MED	0	-	-
		24	$2.35 \times 10^{-4} \pm 4.08 \times 10^{-5}$	$1.68 \times 10^{-3} \pm 5.92 \times 10^{-4}$
		36	$1.68 \times 10^{-4} \pm 2.58 \times 10^{-5}$	$1.71 \times 10^{-3} \pm 5.12 \times 10^{-4}$
		48	$3.21 \times 10^{-4} \pm 3.96 \times 10^{-5}$	$1.09 \times 10^{-3} \pm 1.02 \times 10^{-3}$
		72	$2.88 \times 10^{-4} \pm 1.05 \times 10^{-4}$	$8.56 \times 10^{-4} \pm 2.75 \times 10^{-4}$
		96	$5.48 \times 10^{-4} \pm 1.22 \times 10^{-4}$ *	$4.45 \times 10^{-4} \pm 1.44 \times 10^{-4}$
TYLCV-Mld	MEAM1	0	-	-
		24	$3.33 \times 10^{-3} \pm 7.50 \times 10^{-4}$	$1.97 \times 10^{-3} \pm 5.80 \times 10^{-4}$
		36	$4.23 \times 10^{-3} \pm 7.83 \times 10^{-4}$	$1.55 \times 10^{-3} \pm 1.62 \times 10^{-4}$
		48	$4.21 \times 10^{-3} \pm 1.07 \times 10^{-3}$	$4.10 \times 10^{-3} \pm 1.26 \times 10^{-3}$ *
		72	$4.49 \times 10^{-3} \pm 1.06 \times 10^{-3}$	$1.19 \times 10^{-3} \pm 2.98 \times 10^{-4}$
		96	$5.59 \times 10^{-3} \pm 7.93 \times 10^{-4}$	$1.80 \times 10^{-3} \pm 3.19 \times 10^{-4}$
	MED	0	-	-
		24	$2.70 \times 10^{-3} \pm 5.45 \times 10^{-4}$	$9.10 \times 10^{-4} \pm 1.50 \times 10^{-4}$
		36	$3.38 \times 10^{-3} \pm 3.34 \times 10^{-4}$	$2.58 \times 10^{-3} \pm 4.30 \times 10^{-4}$
		48	$3.66 \times 10^{-3} \pm 7.12 \times 10^{-4}$	$2.21 \times 10^{-3} \pm 6.05 \times 10^{-4}$
		72	$3.23 \times 10^{-3} \pm 3.16 \times 10^{-4}$	$2.55 \times 10^{-3} \pm 7.66 \times 10^{-4}$
		96	$6.21 \times 10^{-3} \pm 2.96 \times 10^{-3}$	$1.57 \times 10^{-3} \pm 1.33 \times 10^{-3}$

Supplementary Table S2. Study of linear tendency of accumulation of virion sense (VS) or complementary sense (CS) strand DNA molecules in whiteflies during time-course analyses of Middle East Asia Minor 1 (MEAM1) and Mediterranean (MED) *Bemisia tabaci* individuals that were given a 24 h acquisition access period (AAP) on TYLCV strain Israel (IL) (two replicated experiments, a and b) or Mild (Mld) (c and d) virion preparations and then maintained on virus-free artificial diet. CS and VS amounts were calculated from whitefly-DNA extracts (three biological replicates per time point and virus/*B. tabaci* type combination, and DNA extracted from 50 insect individuals per replicate) obtained at 0, 24, 36, 48, 72, and 96 hours after the beginning of the AAP. Quantitative analysis of VS and CS molecules was performed on DNA extracts according to Rodríguez-Negrete et al. (1). Data from insect extracts were normalized to the 18S ribosomal RNA gene. The log transformed data of CS and VS were subjected to one-way ANOVA by time and, then, linear contrasts were calculated (SPSS statistical package v.22); the slope of the linear regression is provided and cases in which statistical significance was detected at P = 0.01 are indicated with an asterisk.

Assay	VS	CS
a TYLCV-IL MEAM1	-0,023	-0,036
a TYLCV-IL MED	-0,129 *	-0,030
b TYLCV-IL MEAM1	0,018	0,030
b TYLCV-IL MED	-0,029	-0,157
c TYLCV-Mld MEAM1	-0,063 *	-0,010
c TYLCV-Mld MED	-0,068	0,009
d TYLCV-Mld MEAM1	-0,198 *	-0,224 *
d TYLCV-Mld MED	-0,410	-0,162

References

1. Rodríguez-Negrete, E., Sánchez-Campos, S., Cañizares, M. C., Navas-Castillo, J., Moriones, E., Bejarano, E. R. & Grande-Pérez, A. A sensitive method for the quantification of virion-sense and complementary-sense DNA strands of circular single-stranded DNA viruses. Sci. Rep. 4, 6438. (2014).

References

1. Rodríguez-Negrete, E., Sánchez-Campos, S., Cañizares, M. C., Navas-Castillo, J., Moriones, E., Bejarano, E. R. & Grande-Pérez, A. A sensitive method for the quantification of virion-sense and complementary-sense DNA strands of circular single-stranded DNA viruses. Sci. Rep. 4, 6438. (2014).
